# Recent Innovations in Peptide Based Targeted Drug Delivery to Cancer Cells

**DOI:** 10.3390/biomedicines4020011

**Published:** 2016-05-26

**Authors:** Yosi Gilad, Michael Firer, Gary Gellerman

**Affiliations:** 1 Department of Chemical Sciences, Ariel University, Ariel 40700, Israel; yosig@ariel.ac.il; 2 Department of Chemical Engineering and Biotechnology, Ariel University, Ariel 40700, Israel; firer@ariel.ac.il

**Keywords:** targeted drug delivery, therapeutic carriers, peptide–drug conjugates, cancer therapy, cancer imaging

## Abstract

Targeted delivery of chemotherapeutics and diagnostic agents conjugated to carrier ligands has made significant progress in recent years, both in regards to the structural design of the conjugates and their biological effectiveness. The goal of targeting specific cell surface receptors through structural compatibility has encouraged the use of peptides as highly specific carriers as short peptides are usually non-antigenic, are structurally simple and synthetically diverse. Recent years have seen many developments in the field of peptide based drug conjugates (PDCs), particularly for cancer therapy, as their use aims to bypass off-target side-effects, reducing the morbidity common to conventional chemotherapy. However, no PDCs have as yet obtained regulatory approval. In this review, we describe the evolution of the peptide-based strategy for targeted delivery of chemotherapeutics and discuss recent innovations in the arena that should lead in the near future to their clinical application.

## 1. Introduction

**Figure 1:**
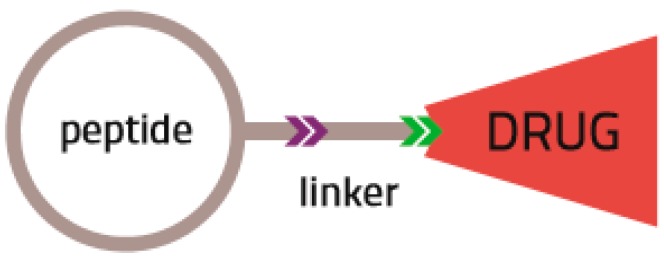
Schematic representation of PDC (peptide based drug conjugate).

Selective delivery of chemotherapeutics to cancer cells has challenged modern chemotherapy from its very beginning. Achieving maximized therapeutic effect as well as minimal off-target side effects requires not only the development of effective strategies for targeted delivery of anti-cancer agents, but also restricted release of the drug payload to the tumor cell or at least its local environment. The strategy of Targeted Drug Delivery (TDD) to tumor tissues is based on the idea that despite the overall molecular similarity between cancer and normal cells, there remains would sufficient cellular heterogeneity to distinguish normal cells from cancer cells. One of the typical biological consequences of cancer is the over expression or unique expression of certain cell surface receptors. These “biomarkers” are often receptors whose activation can lead to enhanced proliferation, migration and invasion of cancer cells and tumor angiogenesis, which are all essential for tumor progression. Selective targeting of these receptors by high affinity biomolecular carriers can mitigate the selectivity problems of chemotherapy [1,2]. Several types of ligands have been tested as potential drug carriers, the most celebrated of which are antibodies [3]. However, there remain a number of pitfalls to their use [3]. In this review, we summarize the strategies, biological activity, challenges and future perspectives of peptide drug conjugates (PDCs) in the targeted treatment of cancer (Figure 1).

## 2. Strategies for Cancer Cell Targeting Peptides Discovery

### 2.1. Phage Display

In 1985, Smith showed that filamentous bacteriophages (bf) can be genetically engineered to express foreign peptide sequences on their surface (protein coat) [4]. This led to the development of libraries of phages in which each clone displays a peptide of combinatorial sequence [5,6]. These libraries can be used *in vitro* to screen for and isolate clones displaying peptides with high selectivity for almost any desired target. They can also be injected into an animal to isolate clones that bind to a desired target tissue [7]. Multiple repetitions on these selection and amplification steps, termed panning, bring about enrichment of the clones with highest affinity to a specific target.

In the literature, there are two approaches to isolating target specific clones; one approach is to expose the total phage pool to the target, for example, an antibody. The binding clones are then recovered and exposed to a non-relevant protein, such as a non-relevant antibody in order to recover only those clones specific to the target antibody’s paratope. The alternate approach proposes that the original phage pool contains mostly non-specific clones that indeed may compete or interfere with the binding of specific clones to the target. Therefore, in this approach, the phage pool is first exposed to one or several non-specific targets and non-binding clones are recovered. These clones are then exposed to the desired target and specific binders isolated.

Since being introduced by Smith [4], this technology—known today as *phage display peptide libraries*—has become a powerful tool for the discovery of specific ligands [6,8] with high receptor affinity [6,9].

Notwithstanding the contribution of phage-display to the discovery of targeting peptides the technique has some critical disadvantages. One of these relates to the method of recovery of phage positive clones. Traditionally, recovered phage are titrated on bacterial lawns, however it is technically challenging to retrieve and sequence the peptide inserts of more than 10–20 such clones. Thus, many potentially interesting clones are missed. The introduction of Next Generation Sequencing (NGS) can now alleviate this problem [10]. While NGS is more expensive and time consuming, it allows analysis of the entire pool of target positive phage. Another disadvantage is that the technology has limited, in that it produces peptides with predetermined length and only from natural amino acids. These disadvantages can be overcome using synthetic combinatorial methods, which are described below.

### 2.2. Synthetic Peptide Libraries—OBOC

In 1984, Geysen *et al.* [11] introduced a combinatorial approach for the segmental epitope mapping of the VP1 protein from the foot and mouth disease virus. A library of 208 overlapping hexapeptides, each peptide differing at one amino acid position, was synthesized, covering the whole 213 amino acid sequence of the protein. The peptide library was synthesized on a solid support, enabling its rapid and feasible immunological screening [11]. In its original form this combinatorial method, later termed “*multipin technology*”, utilized polyethylene pins covered with acrylic acid (for the formation of a polar support with improved solubility in polar solvents) as a solid support. These pins were adapted to a 96 well polypropylene plate, where each well served as a separate reaction vessel. At the end of the synthesis the peptides are directly subjected to a biological screening or are first removed from the pins by enzymatic [12] or chemical [13] cleavage. Several different combinatorial methods and techniques have since been introduced [14,15,16], and the field of synthetic peptide libraries has now become a powerful tool for drug discovery [17,18,19] as well as for fundamental biological investigation [20,21]. In the context of synthetic homing peptides, the one-bead-one-compound (OBOC) method has made a particular impact [22].

The OBOC method is based on a “mix and split” technique and enables preparation of peptide libraries with 10^6^–10^8^ different peptides [23]. The peptides are coated on to 100 µm diameter polymeric beads, each bead covered with about 10^13^ copies of the same peptide [15]. The library can be screened against various biological targets, including intact cells or specific receptors. Beads, which physically bind to the target of interest, are isolated and the structure of the coating compound can then be elucidated [15,24]. Recently a novel screening method for identification of targeting peptides derived from a “mix and split” library was reported [25]. This method involves encoding of each member of the library with a unique peptide nucleic acid (PNA) [26,27] which is biologically stable [28], yet suitable for DNA microarray assays [29,30]. PNA encoded peptide libraries are appropriate for the identification of targeting peptides to any biological target of interest, either *in vitro* or *in vivo* due to the stability of the PNAs in biological environments.

The synthetic flexibility of the OBOC method, and the size of its libraries make it an ideal optimization tool for peptide leads previously discovered by phage display or any other methodology [23].

### 2.3. SPOT-Synthesis

In 1992, Frank introduced a method using cellulose membranes as the solid support for peptide synthesis [31]. In the SPOT synthesis, the peptides are synthesized on pre-functionalized cellulose, which enables the attachment of activated amino acids. “Spotting” small volumes of reagents at defined positions on the cellulose support actually results in a creation of microreactors, whose size is defined by the volumes dispensed and the physical properties of the solid support. The scale of the reaction, as well as the number of synthesized peptides are directly derived both from the size of separate spots on the membrane sheet. Removal of the protecting groups and washings are performed by dipping the sheet in the appropriate solution. After the accomplishment of the synthetic procedure, the peptides can be assayed while attached to the solid support, or can be cleaved for further performance of bioassays in solution. The advantages of SPOT synthesis are that it is a flexible, simple and cheap method, it yields sufficient amounts of product [32], and can be applicable for various biological [33,34] and synthetic [35,36,37] applications. Since the invention of this parallel synthetic technology, it has been developed further by several groups, including the introduction of new polymeric solid supports [38,39,40], linker anchors [35,41,42] and automated systems [42,43].

In contrast to biological combinatorial method such as phage display, synthetic combinatorial methods have the advantage that they can incorporate d-amino acids, unnatural amino acids and non-amino acid building units into the combinatorial sequence [22]. The increased stability of these types of compounds in the proteolytic environment of biological fluids—compared to natural l-amino acids—enhances the half-life of the targeting ligand and can contribute to increased efficacy of the TDD system [44,45,46].

### 2.4. Rationally Designed Peptides

Multistep syntheses and the need for exhausting screening of random combinatorial peptide libraries consisting of millions of different compounds are motivating the design of more target oriented peptide libraries. Rational design of peptide ligands generally depends on bioinformatic databases and a resolved crystallographic structure of the target–ligand complex together with computational methods, in order to design more appropriate binding compounds [47].

One sophisticated approach is based on homology modeling. In this approach the design of new ligands to target is performed by using known targets as structural templates. As we have recently described, the ligand interactions with the different targets can be studied by a stepwise energy evaluation in which the effects of ligand mutations and different residues of the target are examined [48,49]. Such an approach provides a valuable alternative to a costly and time-consuming combinatorial approach since it can dramatically decrease the number of candidate peptides to be synthesized and tested.

**Figure 2:**
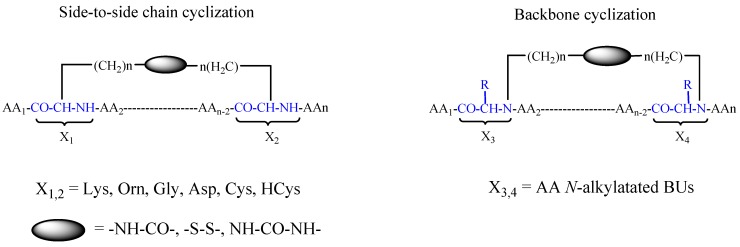
Schematic representation of peptide optimization methodologies.

Rational design of peptides is usually validated by two optimization methodologies: cyclo scanning (CYCLOSCAN) and positional scanning. These methods are also useful in phage display and OBOC for optimizing peptide hits. Notably, classical modes of cyclization include the formation of a lactam bridge through carboxyl and amino functional groups, or disulfide bridges through thiol groups leading to side-chain-to-side-chain bridge formation (Figure 2). Two main drawbacks to this classical mode were reported: (i) cyclization may lead to a loss of biological activity, due to the involvement of side chain groups crucial for bioactivity; and (ii) the number of cyclization possibilities is limited. If the linear peptide does not contain the appropriate amino acid to allow classical cyclization, various amino acids in the native sequence need be replaced by amino acids bearing amine (Lys, Orn), carboxyl (Glu, Asp) or thiol (cysteine), an operation that may lead to loss of biological activity [50,51].

To overcome these pitfalls, Gilon *et al.*, applied a new facile approach of backbone cyclization in which any two backbone nitrogens are connected through bridges of various sizes and chemical nature [52,53,54,55]. In this method, side-chains are not altered, and a highly variable and large set of different cyclizations can be applied to any linear sequence [56]. Thus, one can generate conformational libraries in which many diverse amino acid sequences share the same structure, thereby enabling the optimization of a known three-dimensional biological motif, or libraries in which a single sequence is contained within a large variety of conformations, thereby identifying the active conformation of a biologically active sequence.

Positional scanning for peptide sequence begins with identification of an amino acid of interest at a single position, followed by sequential substitution with other amino acids. Increased bioactivity of the peptide indicates the preferred amino acids at altered positions in the sequence [57,58].

## 3. Targeted Delivery of Chemotherapeutics Based on Clinically Investigated Peptides: Arginine-Glycine-Aspartic Acid (RGD), Somatostatin, Luteinizing Hormone-Releasing Hormone (LHRH) and Bombesin

The success of using peptides to target over or exclusively expressed receptors in cancer cells, including those that are associated with tumor angiogenesis, serves as a basis for the creation of targeted drug delivery (TDD) systems. These systems are generally constructed with a peptide as a targeting moiety, a linker moiety and a therapeutic agent, as schematically presented in Figure 1. While a number of targeting peptides clinical applications have been isolated and are being developed), the tripeptidic sequences—RGD—have received significant attention.

### 3.1. RGD

In their pioneering work in the mid-1980s, Ruoslahti and Pierschbacher reported on the importance of the Arginine-Glycine-Aspartic acid (RGD) tri-sequence in fibronectin as an essential cell recognition site for integrin α_5_β_1_ [59]. This observation has rapidly led to other evidence for the central role of RGD as a general ligand for additional proteins [60,61,62,63,64,65].

In parallel, a family of glycoprotein cell surface receptors was discovered that were recognized by the RGD sequence, [66,67,68,69,70,71,72]. This class of cell surface receptors, consisting of two subunits in mammalian cells [73,74], were for the first time termed “Integrins” in 1986 for their role as “an integral membrane complex involved in the transmembrane association between the extracellular matrix and the cytoskeleton” [75].

Since integrins are involved in processes which are usually associated with tumor progression such as angiogenesis, invasion and metastasis, and since the RGD peptidic ligand selectively targets them, integrins have attracted special focus. Currently, 24 distinct integrins are known [76] and they have been shown to play key roles in many processes including cell adhesion, migration, and proliferation [77]. Enhanced expression of specific integrins in cancer cells is crucial for promoting metastasis [78,79,80], angiogenesis [81], proliferation [82,83], migration [84,85,86] and invasion [87,88], as well as for the proteolytic destruction of extra cellular matrix (ECM) [87], all essential components in the process of tumor progression [89]. The variety of essential functions attributed to the different members of this receptor family in the neoplastic process have been comprehensively reviewed [89,90]. The over expression of integrins and their important role in different cancers, make them an obvious target for therapeutic intervention [91,92], as well as for the selective delivery of chemotherapeutics [93,94,95,96], nanoparticles [97], and imaging agents [98,99]. RGD has for several reasons often been selected as an attractive targeting ligand in many peptide–drug conjugates: it is recognized by 8–12 of the 24 known integrins [93], and there is confirmation of the recognition of RGD by integrins on the structural basis [100], which is also supported by the crystal structure of α_v_β_3_ integrin with the RGD analog Celingetide [101]. For example, Ruoslahti et al published a work in which RGD peptides were used to selectively deliver cytotoxic compounds to cancer cells. The researchers showed that doxorubicin covalently conjugated to the nonapeptide CDCRGDCFC considerably improved survival rates of mice bearing human MDA-MB-435 breast carcinomas [94]. In another paper, Sherz and coworkers reported on selective accumulation of the cyclic RGD analog conjugated to the fluorescent bacteriochlorophyll analog in the tumor necrotic domain in MDA-MB-231-RFP bearing mice, allowing early detection of tumor proliferation [102]. Conjugation of highly potent microtubulin poison paclitaxel to the bicyclic RGD peptide Ec(RGDyK)_2_ resulted in increased drug efficacy towards tumor cells and decrease in off-target toxicity [96].

Other highly effective RGD analogs include 9-RGD [103], iRGD [104], and the cyclic penta-peptide Cilengitide, the latter being developed by Kessler and co-workers [105,106]. Cilengitide has reached phase III clinical trials for the treatment of glioblastoma [107] and phase II clinical trials for some other tumors [92]. In all these cases Cilengitide acts as a highly specific antagonist of α_v_β_3_ and α_v_β_5_ integrins [92,101,107], which are found to be over-expressed integrins in many cancerous cells. To enable conjugation of imaging and therapeutic payloads to Cilengitide, functionally adopted derivatives had been designed [108]. Recently, we reported the synthesis of three novel peptide–drug conjugates based on the cyclic (RGDf(*N*Me)V) penta-peptide in which the methylated valine was mutated to either Lys or Ser enabling primary amine or hydroxy group as a site for drug conjugation [109]. The chemotherapeutic drugs Chlorambucil (CLB) and Camptothecin (CPT) were conjugated to the peptide carrier through amide, ester (for CLB) and carbamate (for CPT) bonds (Table 1). In that report, we provided computational evidence that the conjugation of drug moieties to the backbone of the parent peptide does not significantly alter its conformational space. Thus all conjugates are expected to adopt solution conformations similar to the bio-active conformation of the parent peptide as observed in its complex with the integrin (PDB code 1L5G) [101]. The importance of keeping the correct conformation of the peptide backbone core to maintain binding affinity and receptor specificity was also discussed [92,110,111]. In another report, we described the design and synthesis of four (RGDf(*N*Me)V) based conjugates with dual drug payload, which resulted in enhanced cytotoxic effect towards cancer cells in comparison with mono-loaded counterparts [112]. A similar effect of increased cytotoxicity as a result of multi-loading of the carrier was also observed with a non-RGD peptide-based ligand. In this case, increased loading of the MPB peptide with the alkylating agent CLB resulted in selective enhanced cytotoxicity towards leukemic cells [113].

### 3.2. LHRH

Over expression of luteinizing hormone releasing hormone (LHRH) receptors in hormone-associated cancers makes it another attractive target for a selective delivery of chemotherapeutics. In the mid-1980s, analogs of LHRH peptide were introduced to target LHRH receptor in prostate [114] and breast [115] cancers. Since then this class of peptides has been extensively tested as carriers of chemotherapeutic agents to cancer cells. For example, LHRH analogs were conjugated to doxorubicin (DOX) (conjugate AN-152) or its counterpart 2-pyrollino-DOX (conjugate AN-207), resulting in targeted therapeutic conjugates in various cancer models [116,117,118,119,120,121,122,123,124,125,126,127,128]. In AN-152, the α-keto hydroxyl group on ring A of Dox is linked through an ester glutarate on Lys in position 5 of LHRH. AN-152 (currently named AEZS-108) has reached clinical trials [129,130,131]. Conjugation of LHRH analogs with other toxic agents, such as membrane disrupting peptide [132], toxins [133] and PEGylated delivery systems containing the apoptotic agent camptothecin [134,135], for targeted therapy of cancer cells, were prepared and tested as well (Table 2).

### 3.3. Somatostatin

Somatostatins are a five-membered family [136] of transmembrane G-protein coupled cell-surface receptors widely distributed in a variety of tumors [137,138,139,140,141,142], which also makes them an attractive target for selective delivery of chemotherapeutics. While the native somatostatin peptide has high affinity to all five receptor subtypes, its very short *in vivo* half-life limits its utility as a targeting agent [46]. However, several effective analogs of this peptide have been developed, including two FDA approved and clinically applied radiolabeled conjugates—Octreoscan [143,144,145] and Depreotide [146,147]. Apart from radiolabeled conjugates of somatostatin analogs, several drug conjugates for targeted delivery were reported as well [148]. For example a CPT-somatostatin conjugate, which was developed by Coy and coworkers by directly coupling CPT to the N-terminus of the targeting peptide, showed potent inhibitory activity against various human tumors *in vivo* [149,150,151]. Paclitaxel (PXT) (which interferes with the normal breakdown of microtubules during cell division) was conjugated to the N-terminal of the SST analog octreotide, which resulted in selective activity of PTX towards breast cancer cells MCF-7 [152]. DOX-SST conjugate (AN-238) [153] displayed significant antitumor activity against various cancers including ovarian, prostate, pancreatic, melanoma, lymphoma and glioblastoma [2,154]. Importantly, AN-238 was also able to overcome multidrug resistance induced by conventional chemotherapy [155]. Despite the abundance of reported drug–peptide conjugates in TDD applications, in our opinion the chemistry of carrier–drug attachment has not received enough attention. Characteristics like linker attachment sites that retain carrier activity, linker length and composition, and the design of drug analogs for attachment to the linker are crucial for securing successful drug delivery [156,157,158,159,160]. Redko and co-workers described the synthesis of five novel peptide–drug conjugates based on the disulfide bridged backbone cyclic somatostatin peptide analog 3207-86 which is SSTR2 selective inhibitor [161]. Five chemotherapeutic molecules, acting through different oncogenic mechanisms, were linked to the core peptide carrier, yielding SST–drug conjugates (Table 3). In that work chemo- and biostability of the peptide drug conjugates in various media were measured, representing release profiles for each drug. This information is useful for further optimization of drug release capabilities from 3207-86 peptide–drug conjugates. The 3207-86 cyclic somatostatin peptide was developed by Kostenich *et al.* [162]. In addition, these conjugates were found to be specifically cytotoxic to the cancer cell lines that overexpressed SSTR2, such as HCT 116 human colon adenocarcinoma, H1299 human non-small-cell lung carcinoma and TRAMP C2 human prostate cancer cell lines as oppose to the free drug.

### 3.4. Bombesin

Mammalian Bombesins (BN) are a family of growth receptors (gastrin-releasing peptide receptors (GRPR), neuromedin B receptor (NMBR) and Bombesin receptor subtype 3 (BRS3)) which are frequently overexpressed by a number of common cancers such as prostate, breast, lung, gastric, malignant gliomas and colon [163]. The human GRP, as well as mammalian bombesin—BN receptor ligands are brain–gut peptides—plays an important role in cancer [164,165]. It has been observed that various types of cancers can also synthesize bombesin and GRP [166]. The autocrine mode of action of these peptides brings about stimulation of the growth of the tumor that produces them via bombesin receptors expressed on their surfaces [165,167]. Consequently, BN receptors are interesting targets for TDD to cancer cells. Development of various CPT–BN conjugates has led to the discovery of a potent BN agonist drug conjugate (CTP-L2-BA3) that is cytotoxic for cells overexpressing all mammalian BN receptor subtypes [168]. With the aim of improving the targeting efficacy of the BN–drug conjugate, a multi-ligand approach, whereby paclitaxel (PTX) is conjugated to a divalent BN analog BBN carrier was prepared, resulting in a product with enhanced cytotoxicity [169]. Another BN conjugate was prepared with DOX as a toxic payload [170]. Additionally, cytotoxic conjugates of BN peptides have been prepared by loading them with marine toxins [171], mitochondria-disruptive peptides [172], radiolabeled agents [173,174] and others [175] (Table 4).

### 3.5. Angiopeptin-2

Receptor-mediated transcytosis expedites BBB (Blood-Brain-Barrier) crossing of various macromolecules after initial binding of a targeting molecule to a receptor expressed on brain endothelial cells [176]. Low-density lipoprotein receptor (LDLr) is targeted by angiopeptin-2, defining it as a specific “gate” for delivery of payloads to brain malignancies. The most promising PDC based on this peptide ligand is 19-amino-acid linear angiopeptin-2-paclitaxol PDC (ANG1005) that targets LDLr-1 over expressed on solid tumor [177] and is associated with enhanced transcytosis across the Blood-Brain-Barrier (BBB) [178].

ANG1005 is composed of three molecules of Paclitaxel connected to the two Lys at positions 5 and 9, and to the N-terminal Thr (Table 4). ANG1005 exerts remarkable efficiency in preclinical studies and was well tolerated in phase I clinical studies in glioblastoma. However, phase II clinical trials utilizing ANG1005 were terminated because of lack of efficacy [179]. Other angiopeptide drug conjugates include ANG1007 (angiopep-2–doxorubicin) [180], ANG1009 (angiopep-2–dimethylglycine etoposide), and ANG4043 (angipep 2–trastuzumab). ANG4043 is a novel brain-penetrating peptide–mAb conjugate that is efficient against HER2-positive intracranial tumors in mice (Table 5). This peptide Ab conjugate retains *in vitro* binding affinity for the HER2 receptor and antiproliferative potency against HER2-positive BT-474 breast ductal carcinoma cells [181]. Applications of angiopeptides as targeting moieties for other anticancer applications are still under investigation [181,182,183,184,185].

## 4. Summary and Conclusions

This review has focused on and compared vital parameters of several advanced targeted drug delivery systems. We envisage that these technologies will continue to improve and demonstrate their clinical effectiveness. These technologies are developing in parallel to other drug delivery strategies, which also present technical challenges that need to be overcome. For example, Antibody Drug Conjugates (ADCs) are effective drug delivery systems but present limitations such as complexity of preparation and manufacture, irreproducibility and only modest solid tumor penetrability [205]. Despite these drawbacks ADCs are promising therapeutic modalities and have generated intense interest in recent years. Currently, there are 271 ongoing clinical trials involving ADCs (www.clinicaltrials.gov), suggesting that in the coming years at least several more ADCs will receive regulatory approval. Nanoparticle–drug conjugates (NDCs) are another example. These targeted vehicles successfully extend the circulation time and improve the accumulation and uptake of drugs in tumors due to the Enhanced Permeability and Retention (EPR) phenomenon associated with tumor vasculature. In addition, nanoparticles can be utilized as nano-theranostics, by incorporation of therapeutic and diagnostic agents allowing for simultaneous detection and treatment of tumors [206]. However, NDCs are prone to the same challenges as ADCs, including difficulties in achieving reproducible and controlled synthesis. In addition the EPR effect is unpredictable, and there is currently a lack of a universal standard for evaluating the potent cytotoxicity of NDCs.

Fortunately, PDCs combine the advantages of both ADCs and NDCs and obviate many of their disadvantages. The synthetic and structure–activity relationship strategies mentioned above for rational design and manipulation of the peptide carriers overcome perceived shortcomings of using only linear peptides. These techniques significantly reduce their sensitivity to enzymatic degradation, extensive renal filtration and nonspecific uptake into tissues and organs, all of which contribute to favorable bioavailability and increased half-life in the circulation as compared to other carriers. Another merit of PDCs is their fast and completely reproducible synthesis mostly by solid phase chemistry, enabling their utilization in high throughput screening for rapid optimization of structural parameters. Progress in optimization of “smart” linkers with various activation modes (enzymatic and pH dependent), identification of novel targets and recent discoveries of new peptide carriers for conjugation in TDD [207] will pave the way for greater insight into the contribution of these various characteristics to PDC efficacy, safety and pharmacokinetic properties. Moreover, the development of multi-drug PDCs therapies, namely PDCs that carry cytotoxic “cocktails” instead of single drug, will continue to grow. In conclusion, targeted drug therapies will contribute to major developments in cancer therapy in the near future. ADCs, NDCs and PDCs each have advantages and disadvantages and a better understanding of these will allow a more rational design of combined targeted therapies.

## Figures and Tables

**Table 1:** Luteinizing Hormone-Releasing Hormone (LHRH) cytotoxic analogs.* For additional biological models of these conjugates see [2,154,186].

Sequence of the Targeting Peptide	Name of the Targeting Peptide	Formulation	Therapeutic Agent	Target	*In-Vitro* Model	*In-Vivo* Model	Ref.
GlpHWSYKLRPG-NH2 (Glp = Pyroglutamic acid)	(D-Lys6)LH-RH	PDC	DOX	LHRH	* Human breast cancer cells MCF7 ^a^, Mouse mammary carcinoma cells MXT ^a^, Human breast cancer cells MX-1 ^b^		^a^ [126] ^b^ [118]
GlpHWSYKLRPG-NH2 (Glp = Pyroglutamic acid)	(D-Lys6)LH-RH	PDC	2-pyrrolino-DOX	LHRH	* Human breast cancer cells MX-1 ^a^/ MDA-MB-231 ^b^/ MDA-MB-435 ^c^	* MX-1 ^a^/ MDA-MB-231 ^b^/ MDA-MB-435 ^c^ tumor in mice	^a^ [126] ^b^ [127] ^c^ [128]
Ac-D-Nal(2)-f(4Cl)-D-Pal(3)-SYkLRPa-NH2 [where Nal(2) = 3-(2-naphthyl)alanine, Pal(3) = 3-(3-pyridyl)alanine, and f(4CI) = 4-chloro-d-phenylalanine)	Antagonistic analog	PDC	2-pyrrolino-DOX	LHRH	* Human breast cancer cells MCF7 ^a^, Mouse mammary carcinoma cells MXT ^a^		^a^ [126]
QHWSYkLRP-NH–Et	(D-Lys6)LH-RH Des-Gly10, Pro-NHEt9	PEGylated carrier system	CPT	LHRH	Human ovarian cancer cells A2780		[134]
GlpHWSYKLRPG-NH2 (Glp = Pyroglutamic acid)	(D-Lys6)LH-RH	PDC	Curcumin	LHRH	Human pancreatic cancer cells MIAPaCa-2, BxPC-3 and Panc-1	Pancreas cancer-MIA PaCa-2-tumor in mice	[187]

**Table 2:** Targeted drug delivery (TDD) systems based on Arginine-Glycine-Aspartic acid (RGD) sequence as targeting agent. * For additional examples of RGD–therapeutic proteins conjugates see [188]. ** For additional examples of RGD–nanocarriers targeted therapies see [189].

Name of the Targeting Peptide	Sequence of the Targeting Peptide	Formulation	Therapeutic Agent	Target	*In-Vitro* Model	*In-Vivo* Model	Ref.
RGD4C	Cyclic CDCRGDCFC	* Fusion protein	TNFα	α_V_β_3_	Human glioblastoma cells U87MG, Human breast cancer cells MDA-MB-435, Rat glioma cells C6, Mouse fibroblast cells L929	U87MG tumor in mice, MDAMB-435 tumor in mice	[190]
RGD4C	Cyclic CDCRGDCFC	PDC	DOX	α_V_β_3_		MDAMB-435 tumor in mice	[94]
RGD4C	Bicyclic CDCRGDCFC	Drug conjugate with plasmin self immolative linker vFK	DOX	α_V_β_3_	Human fibroblast cells HT1080, Human endothelial cells HUVEC		[191]
Acyclic RGD4C	Acyclic CDCRGDCFC	PDC	Doxsaliform	α_V_β_3_	Human breast cancer cells MDA-MB-435		[95]
c(RGDfK)	c(RGDfK)	PDC	CLB, CPT	α_V_β_3_	Human non-small lung carcinoma cells H1299, Murine melanoma cells B16-F10, Human embryonic kidney cells HEK-293		[109]
c(RGDfK)	c(RGDfK)	Drug conjugate with dual drug payload	CLB, CPT	α_V_β_3_	Human non-small lung carcinoma cells H1299, Murine melanoma cells B16-F10, Human embryonic kidney cells HEK-293		[112]
c(RGDfK)	c(RGDfK)	PAMAM Drug loaded PEGylated dendrimers	DOX	α_V_β_3_	Human glioblastoma cells U87MG		[192]
c(RGDfK)	c(RGDfK)	PEG polymeric micelles	(DACHPt)	α_V_β_3/5_	Human glioblastoma cells U87MG	U87MG tumor in mice	[193]
c(RGDfK)	c(RGDfK)	** Nanoparticles	DOX	α_V_β_3/5_	Human endothelial cells HUVEC	Pancreas tumor in mice-murine R40P cells	[194]
c(RGDfK)	c(RGDfK)	PDC	CPT	α_V_β_3_	Human prostate cancer cells PC3, Human renal carcinoma cells A498, Human ovarian cancer cells A2780	A2780 tumor in mice	[195]
Celingitide	cyclic-(N-Me-VRGDf-NH)	PDC	Doxsaliform	α_V_β_3_	Human breast cancer cells MDA-MB-435		[95]
c(RGDyK)	c(RGDyK)	Drug loaded PEG-PLA micelles	PTX	α_V_β_3_	Human glioblastoma cells U87MG	U87MG tumor in mice	[196]
c(RGDfS)	c(RGDfS)	PDC	CLB	α_V_β_3_	Human non-small lung carcinoma cells H1299, Murine melanoma cells B16-F10, Human embryonic kidney cells HEK-293		[109]
E[c(RGDyK)]2	E[c(RGDyK)]2	PDC	PTX	α_V_β_3_	Human breast cancer cells MDA-MB-435	MDA-MB-435 tumor in mice	[96]
E-[c(RGDfK)2]	divalent cyclic peptide E-[c(RGDfK)2]	PGA nano-scaled conjugate	PTX		Human glioblastoma cells U87MG, Murine breast cancer cells 4T1, Human endothelial cells HUVEC		[197]
E-[c(RGDfK)2]	E-[c(RGDfK)2]	Peptide drug conjugate with the MMP2/9 sensitive linker GPLGILG	DOX	α_V_β_3_	Human endothelial cells HUVEC, Human ovarian cancer cells OVCAR-3	OVCAR-3 tumor in mice	[198]
E-[c(RGDfK)2]	divalent cyclic peptide E-[c(RGDfK)2]	PDC	PTX	α_V_β_3_	Human endothelial cells HUVEC	ovarian cancer-OVCAR-3-tumor in mice	[199]

**Table 3:** Somatostatin cytotoxic analogs. * For additional biological models of these conjugates see [2,154,186,200].

Name of the Targeting Peptide	Sequence of the Targeting Peptide	Formulation	Therapeutic Agent	Target	*In-Vitro* Model	*In-Vivo* Model	Ref.
RC-160	Cyclic fCYwKVCW-NH_2_	PDC	2-Pyrrolino-DOX, DOX	SSTRs		* MDA-MB-435 tumor in mice, mouse mammary carcinoma-MXT in mice, Dunning AT-1 prostate cancers in rat	[201]
RC-121	Cyclic fCYwKVCT-NH_2_	PDC	MTX	SSTRs		Pancreas cancer-MIA PaCa-2-tumor in mice	[202]
RC-121	Cyclic fCYwKVCT-NH_2_	PDC	2-Pyrrolino-DOX, DOX	SSTRs	* Human gastric cancer cells MKN-45, Human breast cancer cells MDA-MB-231, Human prostate cancer cells-PC-3, Human pancreatic cancer cells-MIA PaCa2, Human SCLC cells H-345	* MDA-MB-435 tumor in mice, mouse mammary carcinoma-MXT in mice, Dunning AT-1 prostate cancer in rat	[201]
3207-86		PDC	Amonafide, ABT-751, CPT, COMB, CLB	SSTR2	Human non-small lung carcinoma cells H1299, Human embryonic kidney cells HEK-293, Human colon cancer cells HTC 116, Human prostate cancer cells TRAMP C2		[161]

**Table 4:** Bombesin (BN) cytotoxic analogs. * For additional biological models of these conjugates see [2,186]. ** For additional BN analogs with 2-pyrrolino-DOX and DOX tested in the same biological models see [170].

Name of the Targeting Peptide	Sequence of the Targeting Peptide	Formulation	Therapeutic Agent	Target	*In-Vitro* Model	*In-Vivo* Model	Ref.
RC-3094	** QWAVGHL–Ψ(CH2-NH)–L-NH_2_	PDC	2-pyrrolino-DOX, DOX	Bombesin	* Human pancreatic cancer cells CFPAC-1, Human lung cancer cells DMS-53, Human prostate cancer cells PC-3, Human gastric cancer cells MKN-45		[170]
RC-3094	QWAVGHL–Ψ(CH2-NH)–L-NH_2_	PDC	2-pyrrolino-DOX, DOX	Bombesin	* Human SCLC cells NCI-H-69	* NCI-H-69 tumor in mice	[203]
BBN(7-13)	WAVGHL-NH_2_	PDC with PEGylated linker	PTX	Bombesin	Human SCLC cells NCI-H-69		[204]

**Table 5:** Angiopep-2 cytotoxic analogs.

Name of the Targeting Peptide	Sequence of the Targeting Peptide	Formulation	Therapeutic Agent	Target	*In-Vitro* Model	*In-Vivo* Model	Ref.
Angiopep-2	TFFYGGSRGKRNNFKTEEY	PDC	3 × PXT	Low-density lipoprotein receptor (LDLr)		U87 glioma	[180]
Angiopep-2	TFFYGGSRGKRNNFKTEEY	PDC	3 × DOX	Low-density lipoprotein receptor (LDLr)	Glioblastoma (U87 MG) Hepatocarcinoma (SK-Hep-1) Lung carcinoma (NCI-H460)	U87 glioma	[180]
Angiopep-2	TFFYGGSRGKRNNFKTEEY	PDC	dimethylglycine etoposide (ETO)	Low-density lipoprotein receptor (LDLr)	Glioblastoma (U87 MG) Hepatocarcinoma (SK-Hep-1) Lung carcinoma (NCI-H460)	U87 glioma	[180]
Angiopep-2	TFFYGGSRGKRNNFKTEEY	Peptide–Ab Conjugate	Trastuzumab	Low-density lipoprotein receptor (LDLr)	HER2-positive BT-474 breast ductal carcinoma cells	HER2-positive intracranial tumors in mice	[181]
